# What Is Mathematical Giftedness? Associations with Intelligence, Openness, and Need for Cognition

**DOI:** 10.3390/jintelligence10040094

**Published:** 2022-10-31

**Authors:** Kaja Hansen, Mieke Johannsen, Laura Langemeyer, Nina Krüger

**Affiliations:** 1 Department Differential Psychology and Psychological Assessment, Institute of Psychology, University of Hamburg, 20146 Hamburg, Germany; 2 Educational Psychology and Personality Development, Institute of Psychology, University of Hamburg, 20146 Hamburg, Germany

**Keywords:** mathematical giftedness, fluid intelligence, openness, need for cognition, adolescents

## Abstract

It is common practice in the educational system to foster high mathematical abilities in schools as well as in specific promotional programs. Still, little is known about the construct of mathematical giftedness itself. In line with intellectual investment theories, our study investigates the relationship between fluid intelligence (figural and numerical), openness, and the need for cognition with mathematical abilities. The current study is based on a sample (*N* = 115) of seventh graders participating in the application process for a promotion program. The results of our regression analyses show a positive link between fluid intelligence and mathematical abilities. However, neither the association with openness nor the need for cognition reached significance, emphasizing the importance of cognitive abilities for mathematical giftedness. Limitations and further directions are discussed.

## 1. Introduction

Individuals differ in their mathematical abilities. While some students perform at low or average levels in this subject, there are some students that exceed expectations and show very high mathematical abilities (Pitta-Pantazi et al. 2011), which is mathematical giftedness. Since mathematical giftedness can be seen as a potential for high performance, there is a need to foster it beyond the usual scholar curriculum (Nolte 2019). While the importance of promotional programs and adequate selection processes through psychological assessments have been addressed (e.g., Benbow 2012; Lohman 2005; Lubinski and Benbow 1994; Stanley 1985), the definition of mathematical giftedness and its interrelatedness with other individual characteristics is still not conclusively clarified in the literature (Bicknell 2009). In line with Kießwetter (1985), mathematical giftedness can be seen as a multidimensional construct that is composed of several traits. Therefore, to assess the mathematical giftedness in students, it is not only important to test their mathematical knowledge but to also consider other relevant constructs such as logical thinking and problem-solving abilities (Fritzlar 2013; Thompson and Oehlert 2010; Warne 2016). However, the use of traditionally widespread selection tools such as intelligence tests in talent promotional programs risks ignoring important aspects of mathematical abilities and thus excluding children that would have been selected through measures of for instance mathematical creativity (Kontoyianni et al. 2013; Lohman et al. 2008). Moreover, the attendance of such programs seems to not only be related to an individual’s cognitive abilities but also to non-cognitive characteristics (Krüger et al. 2019; Meier et al. 2014; Preckel et al. 2008). Since research on associations with related constructs is not conclusive yet, we target the question of whether intelligence and non-cognitive personality traits are related to mathematical abilities in mathematically gifted students.

### 1.1. Mathematical Giftedness and Intelligence

Traditionally, intelligence (IQ) is repeatedly discussed as an indicator of mathematical giftedness (Kontoyianni et al. 2013; Lohman 2005; Warne 2016). Among others, Käpnick et al. (2005) have shown that there are opposing positions on whether intelligence is a sufficient measure of mathematical giftedness. On the one hand, intelligence is highly predictive of school performance, especially grades in mathematics (Lauermann et al. 2020; Roth et al. 2015) and is considered an important indicator of mathematical giftedness by many researchers (Kontoyianni et al. 2013; Lohman 2005; Warne 2016). On the other hand, the mere usage of intelligence as a singular indicator of mathematical giftedness is clearly insufficient (e.g., Kießwetter 1985; Lohman et al. 2008; Nolte 2011). First, the use of general intelligence as a singular criterion does not properly reflect the multidimensionality of (mathematical) giftedness (Kießwetter 1985; Lohman et al. 2008). Second, there is still a considerable proportion of unexplained variance after considering the correlation between intelligence and mathematical performance, indicating the importance of other individual characteristics and abilities for mathematical giftedness (Nolte 2011). More specifically, in a mathematical talent project for primary school students, Nolte (2011) used a common fluid reasoning test, the CFT 20-R (Weiß 2019), to investigate the correlation of intelligence with the selection rank based on the mathematical ability tests. The reasoning score had low predictive power, suggesting that intelligence by itself was an insufficient measure of mathematical giftedness. However, a large proportion of the students that were identified as mathematically talented were also highly gifted (IQ ≥ 130). Thus, a non-negligible variance restriction with respect to the intelligence measure can be assumed and therefore associated statistical limitations such as a decrease in power apply. While the fluid reasoning test of CFT 20-R (Weiß 2019) consists of figural items it also provides an optional number sequence test (ZF) that measures the numerical processing capacity based on numerical material. The ZF therefore can be assumed to be more strongly associated with mathematical performance (Nolte 2011). We will therefore include both the general CFT 20-R and the ZF to investigate the relevance of intelligence and numerical processing capacity for mathematical giftedness.

### 1.2. Mathematical Giftedness and Personality

More recent findings from giftedness research emphasize the relevance of non-cognitive psychological constructs such as personality in terms of the five-factor model (FFM; Costa and McCrae 1992) and the need for cognition (Cacioppo and Petty 1982) in the context of mathematical giftedness (Bruine de Bruin et al. 2015; Colling et al. 2022; Johannsen 2021; Meier et al. 2014; Preckel 2014).

Theoretically, intellectual investment theories (Ackerman 1996; Cattell 1986; Ziegler et al. 2012) emphasize that the acquisition of crystallized intelligence (e.g., cultural knowledge such as mathematical abilities) is facilitated by the investment of fluid intelligence in various learning opportunities. This process is proposed to be influenced by personality variables. The openness-fluid-crystalized-intelligence (OFCI) model (Ziegler et al. 2012) provides a current theoretical framework of how openness, as one of the FFM traits, influences intellectual investment; that is, as individuals high in openness have a wide range of interests and are intellectually curious and imaginative (Costa and McCrae 1992), they are proposed to actively seek intellectually challenging learning opportunities and therefore show higher learning gains and crystallized intelligence. Empirically, openness correlates with intelligence (Costa and McCrae 1992; Lechner et al. 2019; Trapp et al. 2019; Ziegler et al. 2012, 2018) and academic achievement (Poropat 2009). However, the strength of these relations varies across studies and indicators of academic achievement. Therefore, it is of further interest whether openness has an influence on mathematical abilities and modulates the relationship between fluid intelligence and mathematical abilities. First studies suggest that the influence of openness on mathematical skills is rather small (Johannsen 2021; Lechner et al. 2019) compared to other domains. Johannsen (2021) investigated the influence of openness in a sample of mathematically gifted students who had already been accepted and participated in a mathematical promotion program for one to five years. There was no significant correlation between openness and the mathematical test score in the sample. Since all students in the sample had already been selected for the program, the variance can be assumed to be limited. In the current study, we aim to avoid such a restriction by including all mathematically interested students applying for a mathematical promotion program.

Besides openness, another trait of interest is the need for cognition, which might influence how children use their fluid intelligence to acquire more specific skills. Cacioppo and Petty (1982) first conceptualized this construct as a need for cognitive effort and enjoyment of intellectual challenges. In recent years, substantial research has been conducted regarding the relationship between the need for cognition with academic achievement (Colling et al. 2022; Luong et al. 2017; Preckel 2014) and with giftedness (Meier et al. 2014; Schneider et al. 2014). A current study revealed a positive relationship between the need for cognition and various subjects such as mathematics in higher school tracks (Colling et al. 2022). Additionally, the need for cognition correlates with intellectual abilities and openness (Von Stumm 2013). Interestingly, the need for cognition seems to be more closely related to mathematical knowledge (Bruine de Bruin et al. 2015; Preckel 2016), while openness is more predictive regarding verbal indicators of crystallized intelligence (Brandt et al. 2019; Preckel 2016). However, in the sample of Johannsen (2021), the need for cognition was not associated with mathematical abilities beyond fluid intelligence and openness. To gain insight into whether the absence of an influence of need for cognition might have been caused by methodological issues, we further examine this association within a broader sample of mathematically interested students in the current study. As results on both individual characteristics are inconsistent and the relative importance regarding mathematical giftedness remains yet unclear, we include both as potential predictors of mathematical abilities within our study.

### 1.3. Current Study

In the current study, we aim to expand the research on links between intelligence, personality, and mathematical giftedness. Therefore, we seek to avoid variance restrictions as in previous research caused by highly selective samples. Our current sample of mathematically interested students was assessed during the entrance examination of a mathematical talent program and thus includes not only participants of such a talent development program but also those who have not been selected for participation.

Building on previous research on the diagnostics of mathematical giftedness in German primary school-aged children, we examine the use of the CFT 20-R as part of the selection process for a subsequent mathematical promotion program for German secondary school students (seventh grade). Thereby, we are able to add to previous research by assessing the potential contribution of intelligence in the selection of secondary students and evaluation of the previous compensatory selection strategy.

Furthermore, we elaborate on the applicability of the OFCI model for mathematical knowledge in a sample of mathematically interested students. We consequently target the following question: How are mathematical abilities related to intelligence, openness, and the need for cognition? See Table 1 for our hypotheses.

## 2. Materials and Methods

The current study was preregistered at the OSF in November 2021 (https://osf.io/kazxm). It was conducted according to the guidelines of the Declaration of Helsinki. Ethical review and approval were waived for this study because we fully informed the parents and children before participation (no deception), it was no clinical setting, the methods were not invasive and there were no psychopharmacological interventions. Therefore, in line with the standards of the German Research Society (Deutsche Forschungsgesellschaft, DFG), we did not apply to an ethics committee.

### 2.1. Sample and Procedure

In total, 115 adolescents (44.35% female) from Hamburg, Schleswig-Holstein and Lower Saxony participated in the 39th Mathematical Talent Search. This is a sample of mathematically interested students who have been informed by their schools, math teachers, or other sources about the blinded. Most of the participants were at the beginning of the seventh school grade (*M*_age_ = 12.42 years, *SD* = .53).

The data collection was realized on two appointments in September 2021. Table 2 gives depicts the collection procedure. Due to the precautions regarding COVID-19, the assessment took place online. The GSAT-M (German Scholastic Aptitude Test—Mathematics; cf. Wieczerkowski 1989) and the questionnaires were implemented in an online survey using the EFS Survey software (Questback GmbH 2019). The intelligence assessment was presented within the Hogrefe Testsystem (HTS 5; Hogrefe Testsystem 2013). On the first appointment, there were two randomized orders in which the tests and surveys were presented. Half of the children either performed the GSAT-M or the questionnaires first (see Table 2). The intelligence assessment was always the last assignment.

There were no significant differences in the GSAT-M scores between the presentation orders, *t*(113) = −.26, *p* = .79, *d* = .05. In a fixed timeframe of one weekend (Saturday morning until Sunday evening), the students could independently decide when they wanted to start the first assessment. They could take a break of individual length before taking the intelligence assessment. Usually, the HTMB (Hamburg Test for Mathematische Begabung; cf.  Fritzlar 2013) is used as an additional assessment of mathematical abilities. However, the HTMB could not be presented online or taken at home. Therefore, the participants performed a substitute assignment which was completed at home^1^.

### 2.2. Measurement

The following section shortly states the used measures and their reliability estimates within the current study. While internal consistencies were calculated for the used questionnaires, the reliabilities of the ability tests including a speed component were evaluated based on Spearman-brown corrected split-half reliabilities (Bühner 2011). The demographic variables gender and age served as control variables.

#### 2.2.1. Mathematical Abilities

Mathematical abilities were assessed using the German version of the Mathematics part of the Scholastic Aptitude Test (GSAT-M; cf. Wieczerkowski 1989). The original Scholastic Aptitude Test (SAT) is used for a national performance comparison of US-American High School students at college entry level (e.g., Slack and Porter 1980). The Center for Talented Youth at John Hopkins University uses the SAT to identify mathematically and verbally gifted students between 12 and 13 years old (e.g., Benbow 2012). The selection process of our promotion program is based on this conceptualization and was evaluated by an international collegial exchange with this research team. We used two randomly assigned parallel forms of the GSAT-M (version C and D) which both consist of two parts with a total of 60 items that must be answered within 60 min. The GSAT-M was implemented as an online questionnaire using the EFS Survey software (Questback GmbH 2019). The participants were not able to return to a previous part of the test once the time limit for this section had passed. Right answers and omissions scored one and zero points, respectively. Wrong answers deduced the sore by a quarter or a third depending on the number of answer alternatives (5 and 4, respectively). The total score served as the measure of mathematical abilities for all analyses. There were no significant differences between the mean scores of the two test versions, *t*(113) = −1.66, *p* = .10, *d* = .31. The Spearman–Brown corrected split-half reliability was high for version C (*r* = .98) and version D (*r* = .96).

#### 2.2.2. Fluid Intelligence

Fluid intelligence was assessed online using the computer-based version of the CFT 20-R (Weiß 2019) within the Hogrefe Testsystems (HTS 5; Hogrefe Testsystem 2013). The CFT 20-R is a well-established test assessing fluid reasoning ability, one facet of intelligence without societal and cultural knowledge. The short version contains four subtests based on abstract figural material: (a) series, (b) classification, (c) matrices, and (d) topologies. Each subtest has a time limit of three to five minutes. In each of the 56 items, participants must select the appropriate answer from five alternatives. The number of correct answers is summed up to form the total and subtest scores. The Spearman–Brown corrected split-half reliability of the total score was estimated at .84 in our sample. On subtest level, reliabilities were sufficient for (a) series (*r* = .68), (b) classification (*r* = .69) and (d) topologies (*r* = .78), but only marginally satisfactory for (c) matrices (*r* = .52).

In addition, the revised version of the numerical sequence test (ZF) was used to measure the recognition of rules and regularities in simple to complex numerical tasks that is numerical processing capacity. The split-half reliability for the ZF in this sample was sufficient (*r* = .89). Total CFT 20-R IQ scores (*M* = 100, *SD* = 15) based on age-specific reference samples were used as manifest indicators of fluid intelligence. As indicators of the numerical processing capacity, the t-distributed ZF scores were transformed to match the IQ scale (*M* = 100, *SD* = 15).

#### 2.2.3. Need for Cognition

The need for cognition was assessed using an online version of the NFC-Teens (Preckel 2016). The NFC-Teens is a self-report scale consisting of 19 items specifically designed to assess the need for cognition in children and adolescents. Participants rated each item on a 5-point scale ranging from 1 strongly disagree to 5 strongly agree. The online version was implemented with the EFS Survey software (Questback GmbH 2019). Scores were calculated according to Preckel (2016) and then z-standardized for further analyses. The reliability (Cronbach’s alpha) for the need for cognition was satisfactory (α = .89).

#### 2.2.4. Openness

Openness was assessed with the German version of the Big Five Inventory 2 (BFI-2; Danner et al. 2016) within the same online survey created via EFS Survey software (Questback GmbH 2019). The BFI-2 consists of 60 items, each rated on a 5-point scale from 1 strongly disagree to 5 strongly agree. Each of the Big Five personality dimensions (openness, conscientiousness, extraversion, agreeableness, and neuroticism) is assessed with 12 items. Openness scores were calculated according to Danner et al. (2016) and then z-standardized for further analyses. In this sample, the reliability of the openness scores was sufficient (α = .76).

### 2.3. Data Analysis

Data were processed and analyzed within SPSS (IBM Corp. 2019) and R Studio (RStudio Team 2020). Preliminary, descriptive analyses of the data (*M*, *SD*), reliability, and correlation analyses were conducted. In a sample of students with mathematical interest variance restrictions were expected and thus evaluated (cf. Johannsen 2021). Sequence effects for the first assessment date were examined to evaluate the randomization. Moreover, we conducted attrition analyses comparing participants completing all assessments to those who did not participate in the intelligence assessment.

Subsequently, we tested the hypotheses with manifest hierarchical multiple regression models including CFT 20-R scores, openness, and need for cognition scores, as well as the control variables (mean-centered age, gender) as predictors of the manifest GSAT-M score. The standardization of predictors allowed for an easier interpretation and comparison of regression weights. Deviating from the pre-registration, a full information maximum likelihood approach was not implemented because it would only have led to the inclusion of one more case. Instead, missing values were treated with listwise deletion.

First, we estimated a model including only the respective control variables. Secondly, we added fluid intelligence (CFT 20-R scores) and numerical processing capacity (ZF scores) as predictors to test their main effects on mathematical abilities (Hypotheses 1a and 1b). Then, we successively added openness (Hypothesis 2a) and its interaction with fluid intelligence (hypothesis 2b) as additional predictors of mathematical abilities. To test the association of the need for cognition with mathematical abilities (Hypotheses 3a and 3b), we followed a similar procedure: (1) using the model including the control variables, fluid intelligence, and openness as predictors as a starting point, (2) adding the need for cognition as a predictor, (3) adding the interaction between fluid intelligence and need for cognition. Regression models were evaluated by the coefficient of determination *R^2^*, and adjusted *R^2^*s are reported as well. Model comparisons were based on the change in *R^2^*.

## 3. Results

Table 3 depicts descriptive statistics as well as intercorrelations of all variables included in the analyses to test our hypotheses.

The mean fluid intelligence of the sample was 121.70 IQ points (*SD* = 16.22). In comparison to Johannsen (2021), the sample is above average but not far above average (highly gifted) and there is no variance restriction, *χ*^2^(102) = 119.28, *p* = .233. The variance of the sample was restricted in openness, *χ*^2^(114) = 82.59, *p* = .024, and need for cognition, *χ*^2^(113) = 65.81, *p* < .001. The attrition analysis revealed a significant difference in openness between students who participated in the whole study and students who did not complete the intelligence assessment, *t*(22.53) = −2.96, *p* = .007, *d* = .66.

### 3.1. Fluid Intelligence

The first linear regression model including only the control variables as predictors did not reach statistical significance, *F*(2, 98) = .55, *p* = .58, *R*^2^ = .011. Table 4 shows the results of the regression models testing Hypotheses 1a and 1b regarding the associations of fluid intelligence, numerical processing capacity, and mathematical abilities.

The addition of both fluid intelligence and the numerical processing capacity yielded model improvements (see Table 5) and the inclusion of both predictors resulted in the highest model fit, *F*(4, 96) = 10.84, *p* < .001, *R*^2^ = .311. Fluid intelligence, *b* = .24, *p* < .001, as well as numerical processing capacity, *b* = .30, *p* = .001, significantly predicted mathematical abilities. That is, an increase in one IQ point in the fluid intelligence score was associated with a gain of .24 points in the GSAT-M score and an increase in one IQ point in the numerical processing capacity score with a gain of .30 points. Fluid intelligence and the numerical processing capacity explained 11% and 9% of the variance in mathematical abilities in this model.

### 3.2. Openness

Table 5 shows the results of the regression models testing Hypotheses 2a and 2b regarding the associations of fluid intelligence, openness, and mathematical abilities. Neither the addition of openness, *F*(1, 96) = 1.12, *p* = .292, Δ*R*^2^ = .009, nor the addition of its interaction with fluid intelligence, *F*(1, 95) = 2.71, *p* <.103, Δ*R*^2^ = .021, to the model including age, gender, and fluid intelligence as predictors yielded a significant improvement in model fit. The regression weights of openness and its interaction with fluid intelligence did not reach significance. Thus, openness and interaction term did not predict mathematical abilities beyond age, gender, and fluid intelligence.

### 3.3. Need for Cognition

Table 6 shows the results of the regression models testing hypotheses 3a and 3b regarding the predictors’ fluid intelligence, openness, and need for cognition. In addition to the model including the control variables, CFT and openness, neither need for cognition, *F*(1, 95) = .52, *p* = .473, Δ*R*^2^ = .004, nor its interaction with fluid intelligence, *F*(1, 94) = .06, *p* = .803, Δ*R*^2^ = .001, improved model fit. Again, the regression weight of the need for cognition and its interaction with fluid intelligence did not reach significance. Thus, the need for cognition was not related to mathematical abilities beyond, age, gender, fluid intelligence, and openness in our sample.

## 4. Discussion

In our broader sample of mathematically interested students, we found a significant relationship between fluid intelligence, numerical processing capacity, and mathematical abilities. However, for the personality traits of interest, openness, and need for cognition, no significant predictions of mathematical abilities could be shown. Thus, our first hypotheses could be confirmed while the second and third hypotheses were not. In the following, we will integrate our findings with those reported in the literature and evaluate potential limitations in our study design. Finally, implications for the selection of mathematically gifted students for promotional programs will be discussed.

The finding, that fluid intelligence, and numerical capacities significantly predict mathematical abilities, is in line with previous research on the relationship between intelligence and mathematical giftedness (Kontoyianni et al. 2013; Thompson and Oehlert 2010; Warne 2016). Other than in the study conducted by Nolte (2011), our study showed no variance restriction. Overcoming this limitation could explain, why we were able to find the expected results, showing positive correlations between the cognitive constructs. Likewise, the correlations are moderate, stressing that fluid intelligence and mathematical abilities as measured in our study are associated but not identical. Future studies could aim to replicate our finding to expand the knowledge about the role of fluid intelligence in mathematical abilities.

No significant results were found regarding the relationship between openness, its interaction with fluid intelligence, and mathematical abilities. Similarly, Johannsen (2021) reported no significant predictive power of this trait for mathematical abilities in her sample of mathematically gifted students. Although we aimed to avoid variance restriction with our broader sample of mathematically interested students in the selection for a promotion program, this limitation was nevertheless present in our sample. A possible explanation for that could be caused by probable pre-selection within schools and the relatively long application process. As teachers were asked to give the information material only to students who might be interested in the extra-curricular program, possibly only students with high openness were motivated to participate in the first place. Then, the students were provided with preparation materials before the summer break which students with high openness probably have studied in more detail. The variance restriction might have masked a potential impact of openness. Furthermore, our measure for openness, the BFI-2 (Danner et al. 2016), was validated for adults with an age range between 18 and 65 years. Since our younger sample had a mean age of 12.42 years, it is possible that the measurement of the personality trait was not overall valid. Other measures specially designed for children such as the Big Five Inventory für Kinder und Jugendliche (BFI-K-KJ; Kupper et al. 2019) might provide better insights into the relationship between openness and mathematical abilities in seventh graders. Lechner et al. (2019), however, showed an influence between the constructs using the short version of the Big Five Inventory (BFI-10; Rammstedt and John 2007) which was also validated for an adult sample. In another study, the BFI was used to assess personality traits including openness in an adolescent sample with an age range from 13 to 19 years finding an effect of openness on school performance (Negru-Subtirica et al. 2020). Thus, it could be argued that the BFI-2 can be validly applied in younger samples. Then, again, the effects of openness proposed in the OFCI model were rather small (Ziegler et al. 2012). Given our comparably low sample size, effects of such a small magnitude probably could not be detected with sufficient power. Nevertheless, when including openness and its interaction with fluid intelligence in the model, the confidence interval shifts noticeably though not significantly. While this tendency should only be interpreted with great caution, it might hint at a possible small impact of openness on mathematical abilities. Moreover, the interesting exploratory finding of a significant difference in openness between the students participating in the intelligence assessment and those who did not raises further questions about the predictive power of this personality trait for mathematical abilities. Future research might use other measures and a less-restricted samples to investigate this issue.

Finally, the need for cognition and its interaction with fluid intelligence were no significant predictors of mathematical abilities. This finding is in line with the results of Johannsen (2021), but contrary to Preckel (2016) who reported a significant relationship between the constructs. Most recently, Colling et al. (2022) found correlations between the need for cognition and mathematics between a test for mathematical abilities and a rating scale of the need for cognition for children. As for openness, we found a variance restriction despite our broader sample, which again might be due to our recruiting procedure and selection program. In future studies, it is therefore important to overcome this restriction in order to test the predictive power of the need for cognition for mathematical giftedness.

Moreover, there is a more general limiting factor to our research the relatively small sample size. As this study took place within an entrance examination of a mathematical talent promotion program the possible sample size itself was limited. Still, the reported sample was larger than in previous investigations (Johannsen 2021), but nevertheless relatively small. Given the small size, sensitivity power analyses^2^ conducted in G*Power (Faul et al. 2009) revealed that only regression weights or increases in the determination coefficient with an effects size *f*^2^ of .075 or higher could have been detected with sufficient statistical power of .9. Thus, effects of openness and its interaction with fluid intelligence in the magnitude reported by Ziegler et al. (2012) could not have reached significance within our sample. Regarding the effects of openness discussed above, meaning these influences might have not been completely absent in our sample, but rather too small to be detected given the sample size. Nevertheless, the lack of power does not sufficiently explain the absence of the cross-sectional NFC effects considering the confidence intervals of the regression weights here. Nevertheless, the sample offered us the unique possibility to address the relevance of both cognitive (i.e., fluid intelligence and numerical processing capacity) and non-cognitive (openness and need for cognition) characteristics for mathematical giftedness.

## 5. Conclusions

In summary, our results suggest, that in a sample of mathematically interested adolescents fluid intelligence is related to mathematical abilities, whereas openness and need for cognition are not. Since there are limitations in our study, further research is needed before implications for the selection process of promotional programs for mathematically gifted students can be derived. Indeed, the traditional role of intelligence in the assessment of mathematical abilities seems to be justified by our results. Nevertheless, intelligence alone cannot be proposed as a sufficient measure of mathematical abilities. Future research is needed to further disentangle the multiple influences on mathematical abilities.

## Figures and Tables

**Table 1:** Hypotheses of the current study.

Hypotheses	a	b
H_1_	There is a positive association between mathematical abilities and fluid intelligence.	There is a positive association between mathematical abilities and numerical processing capacity.
H_2_	There is a positive association between mathematical abilities and openness.	There is an interaction between openness and fluid intelligence in predicting mathematical abilities.
H_3_	There is a positive association between mathematical abilities and the need for cognition.	There is an interaction between the need for cognition and fluid intelligence in predicting mathematical abilities.

**Table 2:** Procedure of the data collection within the mathematical talent search 2021.

	Order 1	Order 2
1st assessment	GSAT-M ^1^	BFI-2 ^2^
	BFI-2 ^2^	NFC-Teens ^3^
	NFC-Teens ^3^	GSAT-M ^1^
	CFT 20-R and ZF ^4^	CFT 20-R and ZF ^4^
2nd assessment	Open-ended Mathematical task	Open-ended Mathematical task ^1^

^1^ GSAT-M (cf. Wieczerkowski 1989), ^2^ BFI-2 (Danner et al. 2016), ^3^ NFC-Teens (Preckel 2016), ^4^ CFT 20-R and ZF (Weiß 2019).

**Table 3:** Means, standard deviations, and correlations with confidence intervals.

Variable	*M*	*SD*	1	2	3	4	5	6
1. Age	12.42	.53						
2. Gender	.46	.50	−.01 [−.19, .18]					
3. GSAT-M	28.92	9.68	−.07 [−.25, .12]	−.04 [−.22, .15]				
4. CFT	121.70	16.22	−.28 ** [−.45, −.09]	.08 [−.12, .27]	.44 ** [.27, .59]			
5. ZF	120.54	11.60	−.39 ** [−.54, −.21]	−.09 [−.28, .11]	.41 ** [.24, .56]	.46 ** [.29, .60]		
6. Openness (z-standardized)	3.78	.54	−.05 [−.23, .14]	.19 * [.01, .36]	.02 [−.16, .21]	.21 * [.02, .39]	−.08 [−.27, .12]	
7. NFC (z-standardized)	74.98	10.19	−.12 [−.30, .07]	.00 [−.18, .19]	.05 [−.13, .23]	.23 * [.04, .40]	.02 [−.18, .21]	.41 ** [.24, .55]

*Note.* Values in square brackets indicate the 95% confidence interval for each correlation. Gender: 1 = female; ZF = numerical processing capacity; NFC = need for cognition. * *p* < .05. ** *p* < .01.

**Table 4:** Regressing mathematical abilities onto fluid intelligence and numerical processing capacity.

Predictor	*b*	95% CI	*sr* ^2^	95% CI	Fit	Difference
Intercept	29.83 **	[27.19, 32.48]			*R*^2^ = .011	
Age	−1.31	[−4.95, 2.33]	.01	[−.02, .03]	[.00, .07]	
Gender	−1.50	[−5.46, 2.46]	.01	[−.02, .03]	*R*^2^*_adj_* = −.009	
Intercept	−7.12	[−21.47, 7.22]				
Age	1.22	[−2.16, 4.60]	.00	[−.02, .03]	*R*^2^ = .226 **	
Gender	−2.29	[−5.83, 1.25]	.01	[−.03, .05]	[.08, .34]	Δ*R*^2^ = .215 **
CFT	.31 **	[.19, .43]	.21	[.07, .36]	*R*^2^*_adj_* = .202	[.07, .36]
Intercept	−35.46 **	[−56.69, −14.24]				
Age	3.03	[−.34, 6.40]	.02	[−.03, .07]		
Gender	−1.51	[−4.89, 1.88]	.01	[−.02, .03]	*R*^2^ = .311 **	
CFT	.24 **	[.12, .36]	.11	[.01, .22]	[.14, .42]	Δ*R*^2^ = .085 **
ZF	.30 **	[.13, .48]	.09	[−.01, .18]	*R*^2^*_adj_* = .282	[−.01, .18]

*Note.* A significant *b-*weight indicates semi-partial correlation is also significant. *b* = unstandardized regression weights. *sr*^2^ = semi-partial correlation squared. Age was mean-centered; Gender: 1 = female; ZF = Numerical Processing Capacity. ** *p* < .01.

**Table 5:** Regressing mathematical abilities onto fluid intelligence and openness.

Predictor	*b*	95% CI	*sr* ^2^	95% CI	Fit	Difference
Intercept	−9.15	[−23.99, 5.69]				
Age	1.19	[−2.18, 4.57]	.00	[−.02, .03]		
Gender	−1.88	[−5.50, 1.74]	.01	[−.02, .04]	*R*^2^ = .234 **	
CFT	.32 **	[.20, .45]	.22	[.08, .37]	[.08, .34]	Δ*R*^2^ = .009
O	−.96	[−2.78, .86]	.01	[−.02, .04]	*R*^2^*_adj_* = .203	[−.02, .04]
Intercept	−7.96	[−22.74, 6.82]				
Age	.94	[−2.42, 4.30]	.00	[−.01, .02]		
Gender	−1.66	[−5.26, 1.93]	.01	[−.02, .03]		
CFT	.32 **	[.20, .44]	.21	[.07, .35]	*R*^2^ = .256 **	
O	9.39	[−3.23, 22.01]	.02	[−.03, .06]	[.09, .36]	Δ*R*^2^ = .021
CFT:O	−.09	[−.19, .02]	.02	[−.03, .07]	*R*^2^*_adj_* = .217	[−.03, .07]

*Note.* As a comparison model at first the predictors’ age, gender, and CFT were included (see Table 4). A significant *b*-weight indicates semi-partial correlation is also significant. *b* = unstandardized regression weights. *sr*^2^ = semi-partial correlation squared. Gender: 1 = female; Age was mean-centered, O = openness (*z*-standardized). ** *p* < .01.

**Table 6:** Regression mathematical abilities onto fluid intelligence, openness, and need for cognition.

Predictor	*b*	95% CI	*sr* ^2^	95% CI	Fit	Difference
Intercept	−9.80	[−24.78, 5.19]				
Age	1.11	[−2.28, 4.51]	.00	[−.02, .02]		
Gender	−2.02	[−5.66, 1.63]	.01	[−.02, .04]		
CFT	.33 **	[.21, .45]	.23	[.08, .37]	*R*^2^ = .239 **	
O	−.68	[−2.66, 1.30]	.00	[−.02, .02]	[.07, .34]	Δ*R*^2^ = .004
NFC	−.71	[−2.65, 1.23]	.00	[−.02, .03]	*R*^2^*_adj_*= .199	[−.02, .03]
Intercept	−9.72	[−24.79, 5.36]				
Age	1.14	[−2.28, 4.56]	.00	[−.02, .02]		
Gender	−2.03	[−5.69, 1.64]	.01	[−.02, .04]		
CFT	.33 **	[.21, .45]	.23	[.08, .37]		
O	−.64	[−2.66, 1.38]	.00	[−.02, .02]	*R*^2^ = .239 **	
NFC	1.28	[−14.62, 17.19]	.00	[−.00, .01]	[.06, .34]	Δ*R*^2^ = .001
CFT:NFC	−.02	[−.15, .12]	.00	[−.01, .01]	*R*^2^*_adj_* = .191	[−.01, .01]

*Note.* As a comparison model at first, the predictors’ age, gender, CFT, and O were included (see Table 5). A significant *b*-weight indicates semi-partial correlation is also significant. *b* = unstandardized regression weights. *sr*^2^ = semi-partial correlation squared. Gender: 1 = female; Age was mean-centered, O = openness (z-standardized); NFC = need for cognition (z-standardized). ** *p* < .01.

## Data Availability

Data are available from the corresponding author upon reasonable request.

## Notes

1 Therefore, we had to deviate from our preregistration and could not include the HTMB as an additional outcome.

2 We did not conduct post hoc power analyses calculating the empirical power for our analyses as they are a direct function of *p*-values (Hoenig and Heisey 2001).
